# Nanoscale investigations of femtosecond laser induced nanogratings in optical glasses

**DOI:** 10.1039/d3na00748k

**Published:** 2023-11-21

**Authors:** Qiong Xie, Nadezhda Shchedrina, Maxime Cavillon, Bertrand Poumellec, Matthieu Lancry

**Affiliations:** a Institut de Chimie Moléculaire et des Matériaux d'Orsay, CNRS-Université Paris Sud, Université Paris Saclay Bât. 410 91405 Orsay France matthieu.lancry@universite-paris-saclay.fr

## Abstract

Femtosecond (fs) laser irradiation inside transparent materials has drawn considerable interest over the past two decades. More specifically, self-assembled nanogratings, induced by fs laser direct writing (FLDW) inside glass, enable a broad range of potential applications in optics, photonics, or microfluidics. In this work, a comprehensive study of nanogratings formed inside fused silica by FLDW is presented based on high-resolution electron microscopy imaging techniques. These nanoscale investigations reveal that the intrinsic structure of nanogratings is composed of oblate nanopores, shaped into nanoplanes, regularly spaced and oriented perpendicularly to the laser polarization. These nanoporous layers are forced-organized by light, resulting in a pseudo-organized spacing at the sub-wavelength scale, and observed in a wide range of optical glasses. In light of the current state of the art, we discuss the imprinting of nanoporous layers under thermomechanical effects induced by a plasma-mediated nanocavitation process.

## Abstract 2

The intrinsic structure of nanogratings imprinted in silica by femtosecond laser direct writing is composed of light-aligned oblate nanopores. These nanogratings are at the origin of the strong form birefringence exploitable to write various optical devices.

## Graphical abstract

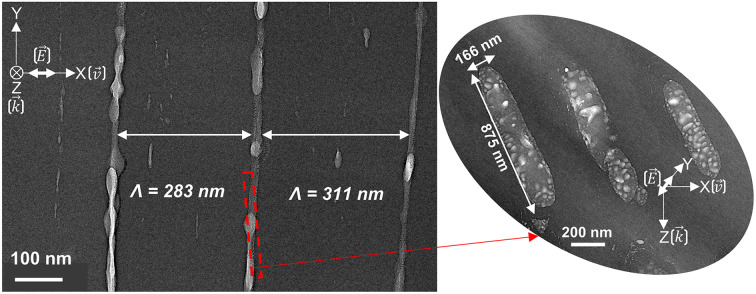

## Introduction

When a femtosecond (fs) laser beam is focused inside a glass material such as silica, the light is nonlinearly absorbed through multiphoton, tunneling and avalanche ionization mechanisms. These complex light/matter interactions lead to the formation of permanent modifications inside, and sometimes around, the irradiated volume. The transformations, while being a function of both glass composition and laser parameters, yield typical transformations classified into regimes, including (i) positive or negative refractive index modifications (commonly called type I),^1^ (ii) formation of volume nanogratings (NGs) (at the root of form birefringence and labeled type II),^2,3^ and (iii) nanovoid-like structures.^4^ The NGs are believed to be the smallest self-organized structures ever created by light in the volume of a transparent material.

The focus of this work is on NGs, which have found use in various fields and applications such as health,^5^ optical data storage,^6–8^ optofluidics,^9,10^ sensors in harsh environments^11,12^ and a wide range of optical components like 3D optical waveguides, 3D geometric phase optics,^13^ and polarizing optical devices.^14,15^ Porous NGs and related strong birefringence are a spectacular manifestation of a light controlled glass decomposition, and have been primarily reported in pure silica^16^ and slightly doped silica glasses.^17,18^ Unlike surface ripples,^19^ NGs were initially found only in a handful of materials: fused silica, sapphire, tellurium oxide, ULE glass and alkali-free aluminoborosilicate glasses.^20–23^ From this list, silica is the material of choice to induce NGs. It brings a wide range of optical functionalities, coupled with high thermal and chemical stability, ease of nanoplasma initiation,^13,24^ and its ability to form nanopores (high viscosity values over a wide temperature range *i.e.* a so-called “long glass”), as opposed to other optical glasses,^25^ thereby offering significant industrial potential as the backbone of many today's photonics applications. Several studies on NG formation were conducted on multicomponent silicate glasses doped with germanium, phosphorus or titanium.^26^ NGs were also found in porous silica prepared from phase-separated alkali-borosilicate glass by removing the borate phase in a hot acid solution.^27^

The importance of understanding NG formation mechanisms lies in the ability to reliably reproduce and potentially scale up the production of NGs, as well as to gain a deeper fundamental understanding of the complex light–matter interactions involved. Numerous research groups have investigated mechanisms behind the formation of self-organized nanogratings, providing valuable frameworks for advancing related scientific inquiries. At the nanoscale, Shimotsuma *et al.*^28^ showed contrast NGs (around 20 nm width and periodicity from 140 to 320 nm) in back-scattered electron imaging corresponding to atomic density contrast. Chemical analysis by Auger spectroscopy revealed that these variations could correspond to oxygen depletion and related density modulation.^29^ Hnatovsky *et al.*^30,31^ reported the presence of nano-cracks and raised questions about whether these NGs can best be described as highly modified regions of differing materials (*e.g.* through bond breaking accumulation) or as some nanovoids. Regardless of the precise mechanistic explanation of nanoplanes (nanoplasma,^32^ photon–plasmons interference,^33^ plasmon–polaritons^34^ or complex self-organization similar to a Turing structure), Lancry *et al.* observed that nanoplanes undergo a glass decomposition coupled with oxygen release.^35^ Asai *et al.* observed a similar feature in GeO_2_ glass^36^ reinforcing the theory of the suggested decomposition process in these oxide glasses. This nanoporosity has been confirmed recently by Richter *et al.* using small angle X-ray scattering,^37^ revealing the formation of elongated nanopores. In 2014 ^38^ and 2018,^19,39,40^ the self-organization process was suggested to be seeded by nanoscale inhomogeneities such as voids and a nanocavitation mechanism was proposed. In 2013, the formation of SiO_2−*x*_ nanocrystals within nanoplanes was reported,^41^ which could be in agreement with oxide decomposition.^42^ In a second publication, the same group did not report nanocrystals but instead revealed that damaged nanoplanes contain randomly dispersed nanopores with a bimodal size distribution.^43^ However, it appears that the use of HF etching degrades the quality of the observations and there are still no reliable nanoscale observations of NGs.

In this paper, we analyze NGs and related nanopore formation inside silica glass using transmission electron microscopy imaging and atomic force microscopy (AFM) techniques. We explored various geometries to probe not only the assembly of nanolayers but also their internal nanostructures. Through high-resolution imaging, we observed a nanoscale assembly of oblate nanopores constituting the nanolayers. Their long axis is found perpendicular to the light polarization while the average periodicity is decreased with the pulse number. These results are then discussed within the framework of a plasma-mediated nanocavitation process and generalized to a wide range of optical glasses.

## Experimental details

The imprinting of NGs in the bulk of fused silica (Suprasil type-I) was performed using a 1030 nm mode locked Yb^3+^ doped fiber laser system (Satsuma, Amplitude Systemes Ltd.). The emitting laser delivered pulses of 250 fs at 100 kHz. Additionally, an aspheric lens (numerical aperture NA = 0.6) was used to focus the laser beam below the sample surface. Due to the minimization of spherical aberration, a laser track was inscribed at a depth of 200 μm. When the laser is translating along *X* and the linear polarization lying along *x*, we define it as “*Xx* writing” (or “*Xy* writing” for a polarization along the *y* axis). Then by moving the sample along the +*X*-axis with a scanning speed of 100 μm s^−1^, a series of adjacent lines being 5 mm long were inscribed. The pulse energy was fixed to either 0.5 μJ or 1 μJ, that is, above the NG formation threshold.^44^ Under these conditions, a strong form birefringence appears due to the presence of porous NGs. For completeness, the optical retardance was measured using a quarter waveplate technique and found to be on the order of 200 nm.^45^

**Fig. 1:**
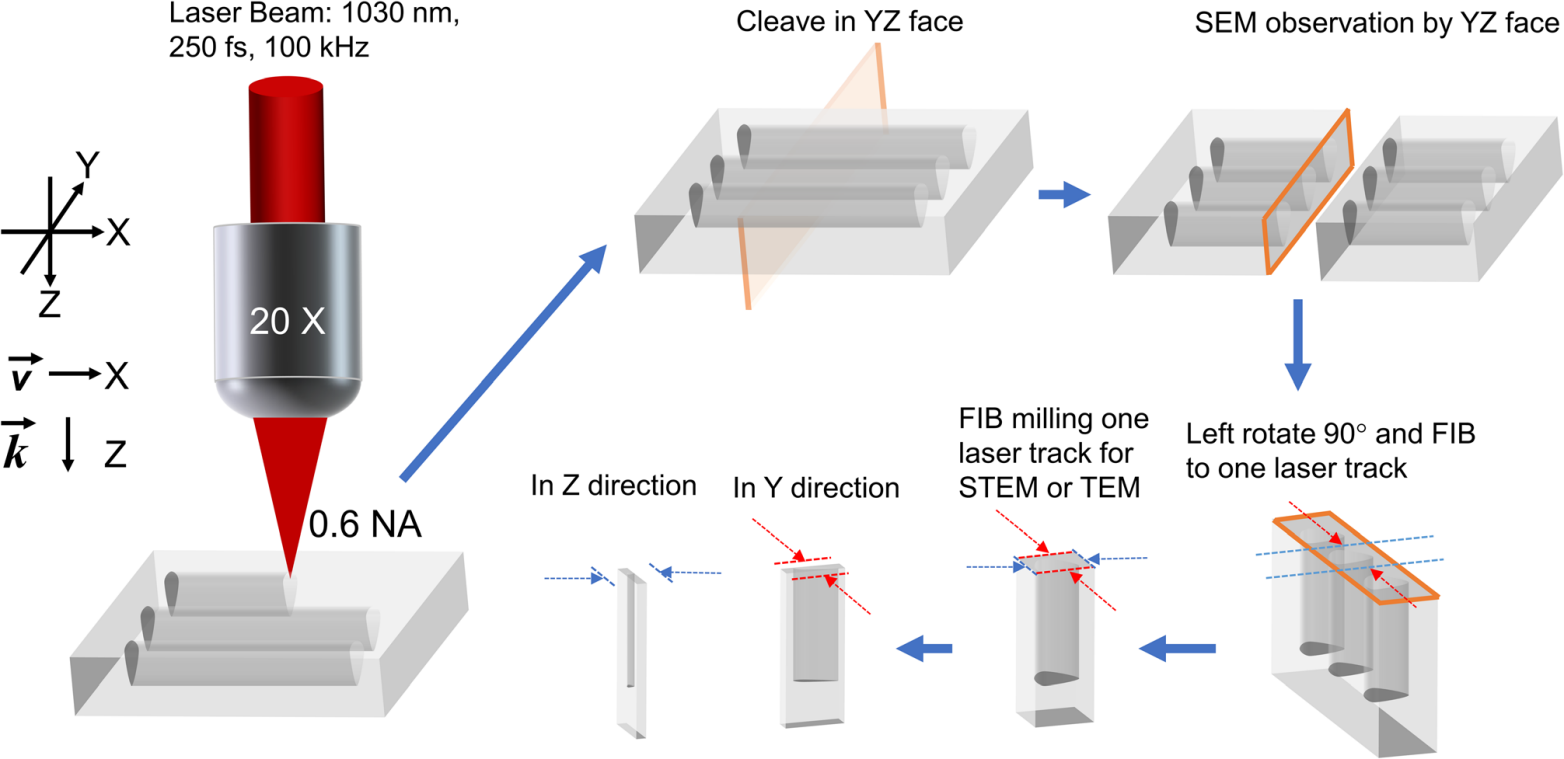
Scheme of FLDW and sample cleaving orientation for subsequent electronic microscopy (SEM, STEM, and TEM) analyses.

To observe the NG nanostructure, each irradiated sample was cleaved using a diamond pen as shown in Fig. 1. Following this, the laser track cross-sections were analyzed by field emission gun scanning electron microscopy (FEG-SEM ZEISS SUPRA 55 VP) for studying the surface morphology. Furthermore, thin samples were prepared using a focus ion beam (FIB) instrument (Zeiss Neon 60, current 50 pA, accelerating voltage 30 kV) to extract slices of NG regions embedded in fused silica glass with a thickness under 50 nm. Here, we used a commercial TOPCON 002B electron microscope (200 kV with a resolution of 0.18 nm). Note that various geometries were employed as sketched in Fig. 1*i.e.*, through transversal (*XY* plane) and longitudinal (*XZ* plane) views.

Finally, scanning transmission electron microscopy (STEM) and high-resolution transmission electron microscopy (HR-TEM) were employed to analyze the internal nanostructures of NGs.

## Results

**Fig. 2:**
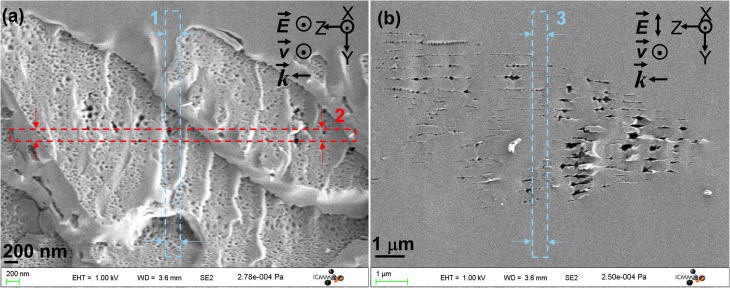
SEM micrographs of (a) nanopores (*Xx* writing) and (b) nanoplanes (*Xy* writing). The red arrows in the *Y* direction and blue ones in the *Z* direction are the directions of FIB milling for TEM sample preparation (typical slice thicknesses are smaller than 50 nm). The numbers 1–3 correspond to the 3 slices extracted by FIB and subsequently observed.

After the FLDW step and before being sliced using the FIB technique, the cleaved samples were observed under a SEM. The resulting cross-sections are shown in Fig. 2 for both *Xx* and *Xy* writing configurations. The darker regions correspond to the nanopores (see Fig. 2(a)) and nanoplanes (see Fig. 2(b)). The gray and white parts correspond to the materials between nanoplanes and nanopores.^46^Fig. 2(a) corresponds to the inside of the nanoplanes that reveal a nanoporous material due to ultrafast oxide decomposition.^46^ In addition, Fig. 2(b) exhibits the sub-wavelength periodicity of the nanoplanes (around *λ*/2*n* with *λ* and *n* being the laser wavelength and the glass refractive index, respectively) along the laser track cross-section and oriented perpendicularly to the laser polarization orientation *Y*. The non-uniformity of the nanoplanes visible from Fig. 2(b) is detailed and discussed in the later analysis of TEM and STEM imaging.

**Fig. 3:**
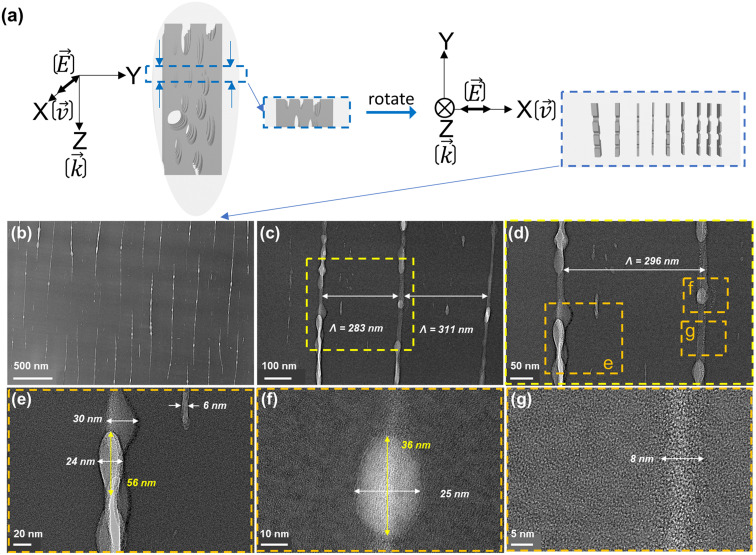
(a) Geometry of the *XY* slice extracted by the FIB milling process. (b–g) TEM micrographs from Fig. 2(a) in blue arrows and *XY* plane observation with different size magnifications. The writing configuration is *Xx*.

To observe the NGs in the transverse view (*i.e.*, in the *XY* plane as indicated by the blue rectangles from Fig. 2), the samples were prepared using a FIB milling process as already described. The resulting morphology of the nanoplanes within the *XY* plane is shown in Fig. 3, with Fig. 3(a) serving as a guide for the reader. In these TEM micrographs, the bright regions are attributed to the fluctuations of the effective electronic thickness of laser-irradiated SiO_2_. The bright lines are thinner regions and likely correspond to the nanoplanes and nanopores. The dark regions correspond to the material situated between nanoplanes. From **E** or **v** (*X*) direction, the nanoplanes exhibit an average period, labeled *Λ*, of 297 ± 14 nm (Fig. 3(c) and (d)). We observe that the nanoplanes are not perfectly aligned perpendicular to **v** or **E** and can present some tilt or wavy appearance. The white porous parts of the nanolayers appear to be several hundred nm long, discontinuous but connected by lamellas of weaker density along *Y* (perpendicularly to the polarization direction). In Fig. 3(e)–(g), the nanostructures are displayed with higher magnification. Nanopores clearly appear as the brightest part of the nanoplanes. They are aligned along each other but quite distributed in size and merging into some whiter matter lamellae like in Fig. 3(e). Based on TEM micrographs of Fig. 3(e)–(g), the nanoporous layer thickness exhibits variable values, averaged to 18 ± 12 nm. The size variation of the nanopores along the *Y* direction ranges from 36 to 56 nm. These observations are consistent with SEM observations in Fig. 2(a), and in agreement with the literature.^46^

**Fig. 4:**
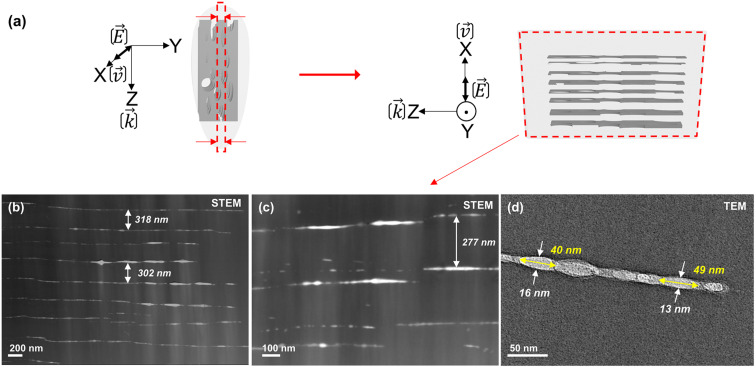
(a) Schematic of the FIB milling process for extracting the *XZ* slice (see red arrows in Fig. 2(a)). (b and c) STEM and (d) TEM micrographs. The writing configuration is *Xx*.

In the aforementioned representation where **E** is parallel to **v**, the nanoplanes are thus aligned along **k**(*Z*) and *Y* directions in SEM or TEM observations. Now by selecting an additional slice by FIB milling identified by the red selection in Fig. 2(a), observations in the longitudinal view (*i.e.*, *XZ* plane) become possible. Corresponding micrographs are provided in Fig. 4(b)–(d). Fig. 4(a) serves as a guide for the reader. Along the *X* direction, which corresponds to either the **E** or **v** direction, the measured period *Λ* and thickness of the observed nanoplanes are around 296 ± 20 nm and 14 ± 2 nm, respectively. The observed nanoplanes showed discontinuities such as partially formed in the **k**(*Z*) direction and it appears pseudo-periodic as first observed in Fig. 2(b). Additionally, from Fig. 4(d) one can observe that both the shape and contrast of the nanoplanes are not homogeneous. The brighter parts correspond to more nanopores superimposed, or larger nanopores, and this is schematically visualized in Fig. 4(a). These overall nanoscale observations align with SEM images in Fig. 2(b), which contain nanopores and with a periodicity along *Z*. This process was attributed to an exciton–polariton-mediated light-organization effect in glass similar to the exciton pattern formation effect observed in cold exciton gases.^34^

**Fig. 5:**
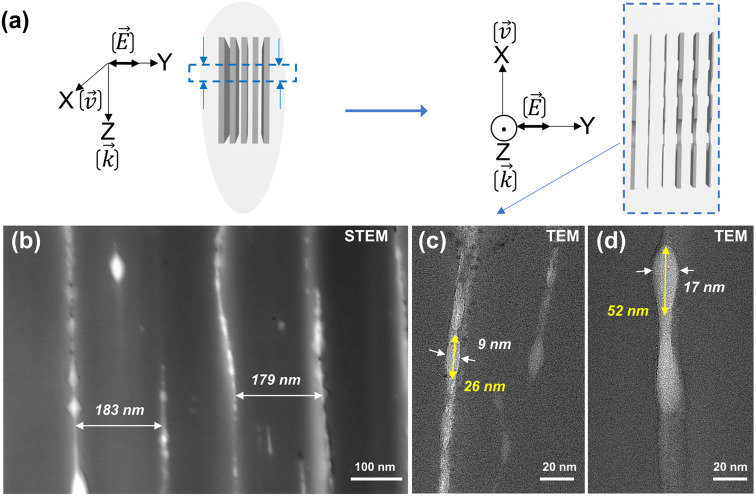
(a) The schematic of the FIB milling process. (b) STEM and (c and d) TEM micrographs from Fig. 2(b) (nanoplanes) in blue arrows in *XY* plane observation. The writing configuration is *Xy*.

Now, changing again our point of view, the results in Fig. 5 represent the *XY* face by taking a sample slice along the blue arrows as sketched in Fig. 2(b). In this configuration, the nanoplanes are aligned along **k**(*Z*) and **v**(*X*) directions. In the **E**(*Y*) direction, the period and thickness of the nanoplanes are respectively 181 ± 4 nm and 13 ± 4 nm. The assembly of subwavelength nanolayers has a shorter period than for *Xx* configuration in agreement with the literature.^47^ However, the dimensions of the nanopores are similar to the one observed for the *Xx* configuration. The nanoplanes exhibit a wavy shape along the **v**(*X*) direction as observed in Fig. 3 and 4.

**Fig. 6:**
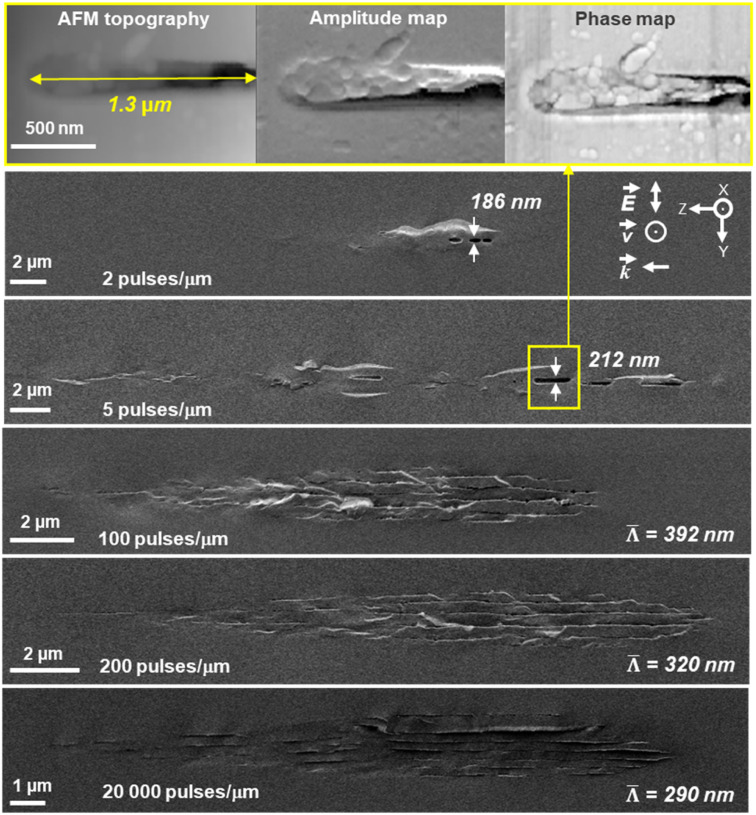
AFM intermittent contact mode and SEM secondary electron images of laser track cross-section according to pulse density expressed in pulses per μm. The laser parameters were: 0.5 μJ per pulse, 1030 nm, 300 fs, 0.6 NA, 100 kHz and *Xy* configuration.

To investigate NG formation in a multipulse regime, we investigated by SEM the step-by-step nanoscale modifications when pulse density is progressively varied from 1 to 2 × 10^5^ pulses per μm. The resulting SEM micrographs are provided in Fig. 6 and highlight the transformation morphology occurring for a perpendicular writing configuration (*i.e.*, *Xy*). The pulse energy was fixed to 0.5 μJ. At a pulse density of 1 pulse per μm, only isotropic index changes are detected by optical microscopy and SEM reveals no specific nanostructure other than a slight contrast related to volume change. At lower pulse densities, between 2 and 10 pulses per μm, we detect a topographic contrast and interestingly some kind of nanopores that evolve into elongated ones (1–2 μm in length and 180–220 nm wide) as pulse density increases. The upper part of Fig. 6 shows atomic force microscopy (AFM) images obtained using intermittent contact mode. Besides the conventional surface topography image (left inset), the cantilever is driven close to a system resonance, to give reasonable amplitude for the oscillation and also to provide phase information, as shown in the right inset of Fig. 6. In particular the phase signal is sensitive to properties of the tip–sample interaction, and may reveal “mechanical information” about the surface such as elasticity, viscosity or adhesion. The observations reveal that the single elongated nanolayer seen below on the SEM micrograph is effectively made of an assembly of nanopores.

**Fig. 7:**
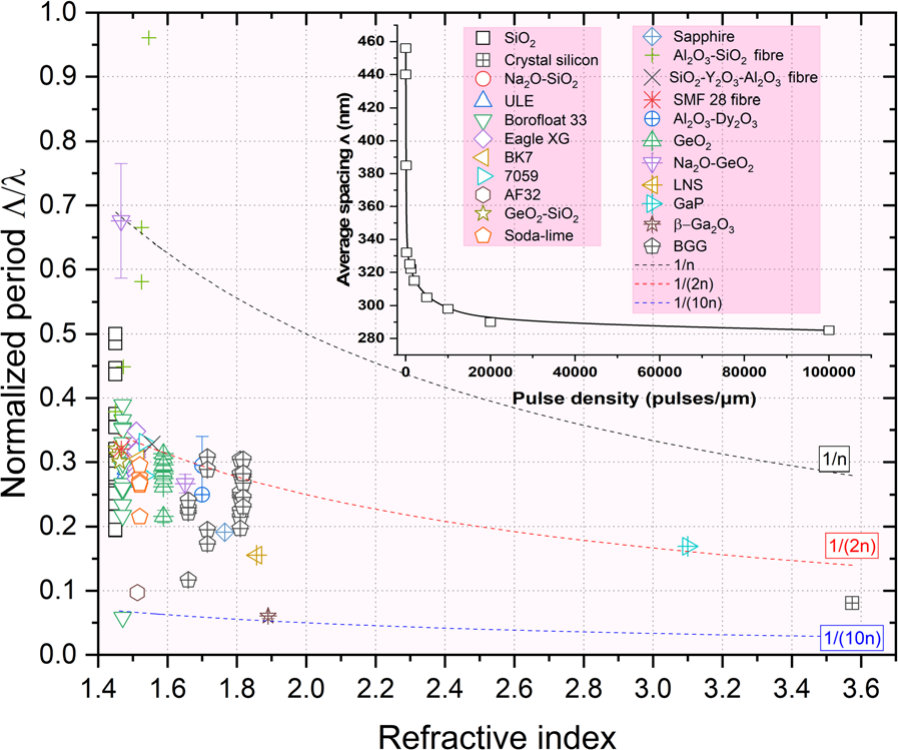
Normalized period *Λ*/*λ* of the overall investigated glasses in the literature including commercial glasses *vs.* refractive index, *n*, measured at 550 nm. The laser wavelength *λ* used in the various experiments was typically between 515 and 1030 nm. Data were extracted from the following ref. 17, 20, 23–26, 45, 48 and 54–67.

At higher pulse densities, these nanopores merge, thus creating some apparently long and thin (typ. 20–30 nm) nanolayers in agreement with the above HR-TEM results. Finally, as the number of nanolayers increases, their average spacing *Λ̄* decreases for pulse densities higher than 100 pulses per μm and reaching up to 2 × 10^5^ pulses per μm in agreement with the literature.^48^ Such quantitative evolution measured in SiO_2_ is shown in the inset of Fig. 7.

## Discussion

From the above HR-TEM and STEM imaging analysis, we can probe the 3D nanostructure of fs-imprinted NGs in silica. We can observe an array of oblate nanopores, which have a long axis oriented both along **k** and perpendicularly to **E**. The thickness of the nanolayers ranges from 6 to 30 nm and their extension perpendicularly to the laser polarization could reach a few μm or more. As shown in Fig. 6, under a multipulse regime, these nanolayers are made of oblate nanopores that obviously “self-align” along each other to create these nanoplanes over quite long distances, as observed in the literature over the last two decades.^28^ We can also observe a subwavelength ordering resulting in an average periodicity, perpendicular to **E**, on the order of 290–390 nm depending on the writing configuration.

Based on these results and on the reported mechanisms of the NG formation in the overall literature, we suggest that NGs are imprinted through a plasma-mediated nanocavitation process with a spatial ordering due to scattered wave interference,^38,49^ which is described below.

The first step would be that some inhomogeneities of dielectric constant seed the process. These inhomogeneities (or seeds) could either be already present in the pristine glass or be photo-induced by the first pulse(s). Following these first instants, a spherical nanoplasma forms, stimulated by plasma density or temperature and evolves into an oblate-shaped nanoplasma over several pulses. This plasma-mediated process has been suggested in the literature. For example, Taylor R. *et al.*^50^ suggested in 2008 that the presence of defects or color centers might seed the plasma, creating locally and easily ionized “nanospots” creating high plasma density. In the model developed by Bhardwaj *et al.*,^51^ the period *Λ* of nanogratings was assumed to remain between *λ*/*n* and *λ*/2*n* and a period initially to be independent of pulse energy. However, the period was shown to decrease continuously with the number of laser pulses^52,53^ but also when playing with the chemical composition such as doped SiO_2_ (ref. 26) or in multicomponent glasses.^23,24^ This is clearly illustrated in Fig. 7 where we summarized the normalized period *Λ*/*λ vs.* refractive index reported in the overall literature including a wide range of commercial glasses.

In 2014 Buschlinger R. *et al.*^38^ conducted finite-difference time-domain (FDTD) modeling of plasma spatial structuration to investigate the periodicity of the NGs. These originated from the randomly distributed nanometer-size inhomogeneity that seeded the plasma structure. Due to the interference between scattered and incident light, the plasma owns a spatial structuration and grows against the direction of the light polarization. In 2016 A. Rudenko *et al.*^68^ developed a numerical model to explain the formation of periodic volume NGs from random inhomogeneities with varying concentration and laser parameters. The contribution of an interplay of the physical processes (*e.g.*, the interference between the incident and the scattered waves, multiple scatterings, local field enhancement, and accumulation processes driven by multiphoton ionization) reinforced in the formation of NGs. Importantly, the period of NGs was found to scale down with growing pulse number, which in turn relies on scattering originating from nanoscale inhomogeneities. Following the above views, we suggest that nanogratings are not self-organized or self-assembled through diffusion-reaction mechanisms like Turing structures but rather “forced aligned” by the light pattern itself. In addition, the authors suggested the presence of some kinds of nanovoids, 0.6 nm average diameter, that are initiating the process. However, what seeds the process is not yet fully elucidated. It could be point defects (like self-trapped excitons (STEs) or self-trapped holes (STHs)), where the valence band possesses high energy and can easily be excited, some voids generated by the first pulse or even some glass-free volume that is already “available” in the pristine glass. Moreover, it exhibits a quite regular structure in the short range, with some *n*-membered rings creating well-known porosity at the sub-nm scale.^69^ This is even reinforced in nanoporous sol–gel silica where the pulse number needed to imprint NGs was observed to be smaller,^70^ when increasing the glass free volume.

From Fig. 3(c) and 4(c), we can observe some short (typ. <50 nm) but always oblate nanopores (with a long axis along **k** and perpendicular to **E**) with a thickness much smaller than 5 nm. They appear to be dispersedly distributed, but sometimes start to align along each other. When increasing the number of pulses, we can expect having more seeds (*e.g.*, some first nanopores or some STH) generated by the first pulses. This, in turn, leads to additional scattering centers resulting in a smaller periodicity based on the scattered wave interference model suggested by A. Rudenko *et al.*^68^ and in agreement with F. Zimmermann *et al.*^48^ who reported a decrease in NG period. It's worth mentioning that there are many “small size nanopores” between the long and well-arranged ones revealing the emergence, and growth, of new nanolayers like in Fig. 3(c) and 4(c). These are some kinds of “seeds” that will grow and merge (see Fig. 6) with neighboring nanopores to become a new porous nanolayer when increasing the number of pulses. According to the suggested “memory effects” involved in the mechanism of nonlinear ionization, new inhomogeneities are generated from pulse to pulse resulting in additional multiple scattering thus organizing the plasma distribution. It assumes that the pulse density is proportional to the concentration of the inhomogeneity.^68^ As the pulse density increases, new plasma nanoplanes are generated “here and there” between the pre-existing ones following the light pattern. The imprinted nanolayers are initially made of elongated nanopores that grow from pulse-to-pulse and merge resulting in new nanolayers. Finally, from pulse to pulse, this will lead to a reduction of the average spacing *Λ̄* of the nanogratings as observed in the inset of Fig. 7.

The second step would be plasma formation and the local field enhancement resulting in ellipsoid (oblate) nanoplasma hot spot (high electron plasma density or energy) formation. Indeed, and even for a single pulse, the disordered spherical nanoplasma would evolve into an ellipsoidal shape and become oblate perpendicularly to the laser linear polarization. This occurs because the laser electric field triggers an asymmetric growth of the nanoplasma.^50^ In contrast, for a circular polarization the nanoplasma hot spots should remain symmetric, resulting in spherical nanopores as has been observed in the type X regime.^13^ Then in a multipulse view, there is an evolution of the oblate nanoplasmas into nanoplanes as shown in “post-mortem” experiments in Fig. 6.^53^ The lengths of the oblate voids, whose direction is perpendicular to **E**, increase as the pulse number increases in agreement also with observations made in the type X regime.^13^ From Fig. 4 one can also notice that such step by step nanopore growth also creates a longitudinal component since nanopores are also “elongated” along the laser beam direction (**k**) and self-aligned to form nanoplanes.

The third step would be the energy transfer between electrons and phonons resulting in localized heat distribution at the nanoscale. Indeed, the modification of transparent glasses with wide bandgap dielectrics is induced rapidly by fs laser pulses through a multiphoton ionization (MPI) within a few femtoseconds. The electron plasma produced by MPI heats the media by electron–lattice coupling. Here we first assume no heat accumulation either considering a single pulse or multiple pulses in this process. The low heat capacity of electrons allows them to be easily heated to extremely high temperatures, but the glass matrix (the lattice) initially remains “cold” due to the relatively long electron–phonon relaxation time, typically 10 ps in SiO_2_. After this time, the lattice will finally heat up, by a few 1000s of °C but this is a local effect. Indeed, after the free electron plasma energy is transferred to the lattice, the spatial distribution of the temperature is quite the same as the one of the plasmas because the timescale (<1 nanosecond) is too short to have some significant heat diffusion (on the order of the μs). In that sense, this is a plasma-mediated process, and the temperature distribution is the image of plasma 3D nanostructuration or the plasma “map”.^46^

The fourth step would be the strain creation due to the temperature difference, between the nanoplasma hot spot and the background, resulting in a local thermal expansion. While the heat diffuses inside the material, the silicon–oxygen bonds would elongate and the glass specific volume expand within a time scale shorter than the characteristic acoustic relaxation time (typ. 500 ps in silica glass)^25^ thus creating a moderate shock wave. So at a short time scale, there exists a localized nanostrain, which distributes ellipsoidally as a mirror image of the temperature map. This in turn will initiate the nanocavitation process at these specific locations. Indeed, a decrease of the local pressure would be created due to the formation of a rarefaction zone behind the “shock wave”. Once this “negative pressure” difference develops between the “pore nuclei” and surrounding materials,^46^ nanopores are imprinted at the image of the plasma ellipsoidal nanostructuration where the nanocavitation process starts. Finally, the formation of nanopores was observed in most oxide glasses^20,24,25,63,66,71–73^ thus revealing that the glass oxide decomposition process occurred in all these compositions, highlighting in such a way that this is a general mechanism.

In this process it seems that nanopores may not have the chance to grow from a spherical shape but rather ellipsoid like the nanoplasma distribution itself for a linear polarization whereas a circular polarization would induce substantially spherical nanopores and thus no/low birefringence.^74^ Following this view, type X is in fact the early birth of nanograting formation mostly observed for a low number of pulses and low energy. These type X modifications refer to oblate (for linear or elliptical polarization) nanopores with low birefringence and ultralow optical losses.^13^ In this mechanism, the small nanopore diameters that are quite randomly arranged result in a decrease of the Rayleigh scattering and thus low optical losses offering exciting prospects for applications. For example, these type X modifications were exploited for achieving a 5D optical storage with high data capacity and long lifetime in fused silica^75^ or to imprint ultralow loss 3D geometric phase optics.^13^

## Conclusions

We investigated some nanoscale aspects of the formation of self-assembled porous nanogratings in oxide glasses. Oblate nanopores populate some array of non-continuous nanoplanes, which grow perpendicularly to the laser polarization direction and along the laser propagation direction. Some tiny elongated nanopores were also found between the long and fully-grown nanoplanes. These nanopores will grow and merge in a multipulse regime, resulting in a pulse-to-pulse decrease of the average periodicity much below *λ*/2*n* as reviewed in this paper. The plasma-mediated nanocavitation model discussed the formation of these “light forced-organized” (rather than self-organized) sub-wavelength NGs in a multipulse view. Our tentative interpretation supported by HR-TEM and STEM investigations proposes an overall framework for NG formation. This mechanism is useful to guide future experiments to explore the interaction between laser and optical materials, along with enabling one to better control NG formation and its generalization in any kind of optical glasses.

## Author contributions

Conceptualization, M. C. and M. L.; funding acquisition, B. P., M. C. and M. L.; investigation, Q. X., N. S.; methodology, Q. X., M. C. and M. L.; project administration, M. L.; resources, M. L.; supervision, M. L.; validation, Q. X.; visualization, Q. X. and M. C.; writing—original draft, Q. X.; writing—review & editing, Q. X., N. S., M. C., B. P. and M. L. All authors have read and agreed to the published version of the manuscript.

## Conflicts of interest

There are no conflicts to declare.

## Supplementary Material
